# An Overview on Single-Cell Technology for Hepatocellular Carcinoma Diagnosis

**DOI:** 10.3390/ijms23031402

**Published:** 2022-01-26

**Authors:** Sheik Aliya, Hoomin Lee, Munirah Alhammadi, Reddicherla Umapathi, Yun Suk Huh

**Affiliations:** Department of Biological Sciences and Bioengineering, NanoBio High-Tech Materials Research Center, Inha University, Inha-ro 100, Incheon 22212, Korea; biotechaliya@gmail.com (S.A.); hmlee8907@gmail.com (H.L.); munirahabdulaziz00@gmail.com (M.A.); umapathi4u@gmail.com (R.U.)

**Keywords:** hepatocellular carcinoma, liver cancer, RNA sequencing, single-cell technology

## Abstract

Hepatocellular carcinoma is a primary liver cancer caused by the accumulation of genetic mutation patterns associated with epidemiological conditions. This lethal malignancy exhibits tumor heterogeneity, which is considered as one of the main reasons for drug resistance development and failure of clinical trials. Recently, single-cell technology (SCT), a new advanced sequencing technique that analyzes every single cell in a tumor tissue specimen, aids complete insight into the genetic heterogeneity of cancer. This helps in identifying and assessing rare cell populations by analyzing the difference in gene expression pattern between individual cells of single biopsy tissue which normally cannot be identified from pooled cell gene expression pattern (traditional sequencing technique). Thus, SCT improves the clinical diagnosis, treatment, and prognosis of hepatocellular carcinoma as the limitations of other techniques impede this cancer research progression. Application of SCT at the genomic, transcriptomic, and epigenomic levels to promote individualized hepatocellular carcinoma diagnosis and therapy. The current review has been divided into ten sections. Herein we deliberated on the SCT, hepatocellular carcinoma diagnosis, tumor microenvironment analysis, single-cell genomic sequencing, single-cell transcriptomics, single-cell omics sequencing for biomarker development, identification of hepatocellular carcinoma origination and evolution, limitations, challenges, conclusions, and future perspectives.

## 1. Introduction

An estimated 844 million people worldwide are affected by liver disease, of which liver cancer constitutes the most lethal malignancy. Primary liver cancer (starting in the liver) affects nearly 40,000 Americans each year, and men are affected more than women [1]. According to the American Cancer Society, approximately 20,020 men and 10,140 women will die this year due to this severe disease, while it has been reported that nearly 380,000 people in China die every year due to this cancer [2]. The incidence rate of primary liver cancer has tripled since 1980, with just an 18% survival rate while the death rate doubled in 2020 [3]. Among various types of primary liver cancer, the most common is hepatocellular carcinoma (HCC) or hepatoma [4]. Approximately 10–20% of primary liver cancers are bile duct cancers (intrahepatic cholangiocarcinomas). Hepatoblastoma (occurs in children below 4 years old), angiosarcoma, and hemangiosarcoma are rare types of primary liver cancer. Benign liver tumors including hemangioma (starting in liver blood vessels), hepatic adenoma, and focal nodular hyperplasia are difficult to treat but radiation and chemotherapy slow their progression [5].

In the past, liver cancers were screened by the alpha-fetoprotein (AFP) blood test, which failed to detect early-stage cancer. Thus, new blood tests such as DCP (Des-gamma-carboxy prothrombin), osteopontin, Glypican-3, and Golgi protein-73 were developed in addition to imaging tests such as CT (computerized tomography) and MRI (Magnetic resonance imaging) scans for accurate cancer diagnosis. Liver cancer is the second leading cause of death worldwide, ranked as the sixth most frequently diagnosed cancer. A higher rate of deaths due to liver cancer has led to developing novel diagnostic techniques, treatment procedures, and prevention of recurrence after surgery (adjuvant therapy) [6]. The therapies include radioembolization, chemotherapy or chemoembolization, neoadjuvant therapies (treatment before surgery) which include targeted therapy [7,8], viral therapy, ablation therapy (irreversible electroporation), trans-arterial chemoembolization (TACE) [9], and embolization. Even with all these treatment strategies in hand, progressive stages of HCC remain mostly incurable as they are diagnosed at an advanced stage, and a gap remains [4].

Recently, intratumor genomic heterogeneity in several tumor types has been documented with evidence linking it to cancer prognosis [10]. Zheng et al. hypothesized that survival of a tumor heterogeneous community is mainly due to the collective role of each cell which is hierarchically organized in a tumor lesion. However, the existing genomic analyses are not robust enough to describe the true evolution process of tumors and the development of cell communities. It is puzzling to imagine how the heterogeneous tumor cells communicate or regulate or cooperate preparing themselves to survive as an efficient community. Notably, these tumor communities have not been characterized [11]. In HCC, intratumor molecular heterogeneity is attributed to the presence of cancer stem cells (CSCs). CSCs or tumor-initiating cells are a subpopulation of cancer cells that are key responsible cells for tumor initiation and growth maintenance. Each CSC population is defined by specific cell surface markers characterized by different oncogenic drivers, which poses a challenge to current targeted molecular therapeutics. A new technique called single-cell technology (SCT), or single-cell RNA sequencing (scRNA-seq), has filled this gap by enabling the study of single cells at the transcriptional level. This has paved the way for the discovery of altered cell types such as liver progenitor cells and subtypes in diseased liver cancer samples versus normal liver samples. This technique has provided evidence that CSC subpopulations exhibited distinct molecular signatures at the tissue level while, at the single-cell level, they were phenotypically, functionally, and transcriptionally heterogeneous [11]. The difference in gene expression pattern between individual cells of single biopsy tissue critically helps in identifying and assessing rare cell populations which normally cannot be identified from pooled cell gene expression pattern (traditional sequencing technique) [12]. Interestingly, this diversity has proved to be effective in HCC prognosis.

This review consists of nine sections. Section 1 specifically emphasized the SCT, which provides novel insights for understanding cellular heterogeneity in liver cancer or HCC, which in turn assist in the design of accurate treatment strategies. Section 2 provides information on single-cell technology. Section 3 offers information on hepatocellular carcinoma diagnosis. Section 4 deliberates on the tumor microenvironment analysis. Section 5 presents single-cell genomic sequencing. Section 6 introduces single-cell transcriptomics. Section 7 includes details on single-cell omics sequencing for biomarker development. Section 8 encompasses the identification of hepatocellular carcinoma origination and evolution. Section 9 elucidates the limitations and challenges. Section 10 covers conclusions and future perspectives. We anticipate that this comprehensive review will serve as a cornerstone for the future developments of SCT. Furthermore, this review will deliver novel insights on SCT for realizing cellular heterogeneity.

## 2. An Overview of Single-Cell Technology

**Figure 1:**
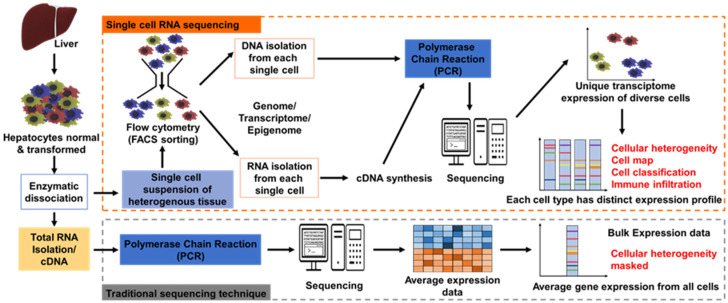
General overview of single-cell RNA sequencing and traditional sequencing techniques.

SCT, otherwise called scRNA-seq, is the sequencing of a single cell genome or transcriptome to get information about genome or transcriptome or other integrative omics (proteome, metabolome, and epigenome) (Figure 1). sc-RNA-seq was first introduced in 2009 by Tang et al. Earlier, this new technology was not highly popular because of cost constraints, and other problematic issues include the presence of a low proportion of the genes, limited coverage of the transcript, and cannot be applied for the frozen tissue samples, but technical protocol modifications brought widespread popularity by 2014. SCT encompasses a two-step process: single-cell separation and single-cell analysis. Single-cell separation is achieved by flow cytometry, laser capture micro-dissection (LCM), optical tweezers, and microfluidics. All these processes form the basis of single-cell analysis, which includes the profiling of specific cell genomes, transcriptomes, and proteomes [13]. SCT has transformed the understanding of disease pathogenesis; subsequently, it describes in detail unprecedented interactions between pathogenic cell populations and the homeostatic environment in the tissue. This offers an advantage over the traditional techniques where profiling of bulk cell population is done. In the conventional approach, tumor cells’ co-occurrence mutation pattern is not resolved, while SCT examines in-depth complexity by identifying compound mutations which are particularly useful to categorize pathways activated and the cause of tumor cell resistance. SCT adds a new dimension to the transcriptomic data by analyzing cellular heterogeneity in tumor or diseased tissue. Cellular heterogeneity of tumor or intratumor heterogeneity (ITH) refers to distinct phenotypes and genetic alterations happening within a single or different tumor nodule (tissue biopsy) of the same patient. Cellular heterogeneity imparts major clinical consequences by misdirecting treatment decisions. However, implying molecular information of single tissue biopsy potentially favors sampling bias and helps in deciding the accurate drug for the treatment of the patient. Apart from cancer biology, single-cell sequencing technology is nowadays applied in various biological studies like microbiology, immunology, reproduction biology, neurology, digestive, and urinary systems. This clarifies its role in basic science and clinical research area [14]. In the near future, millions of cells can be routinely analyzed. Already, a pilot study of the human cell atlas with 35 trillion cells map of the human body has started [15]. Presently, SCT has provided a favorable platform for tumor diagnosis by developing specific tumor biomarkers and individualized tumor therapy [16]. Thus, on a global scale, this technique will bring revolution in the prognosis and treatment of various types of cancer [17]. For instance, SCT has been designated as a robust technique to diagnose (through a noninvasive method) bladder cancer by screening urine for rare malignant cells. This technique surveys malignant cells’ copy number alternations or tumor (oncogenic) driver gene mutations of characteristic cancer [18]. SCT is indispensable for the identification of biomarkers of gastric cancer, lung cancer, colorectal cancer, breast cancer, and various other tumors, thus contributing to early diagnosis and prognostic monitoring of cancer [16]. Recently, this novel approach has been applied in the field of hepatology. Figure 1 provides a generalized overview of single-cell RNA sequencing and traditional sequencing techniques.

## 3. An Overview of Hepatocellular Carcinoma Diagnosis

Worldwide, HCC is a serious health challenge as it has created a hefty economic burden due to increasingly high mortality rates in the general population. At the initial stages of the disease, an HCC patient displays no clinical symptoms, which leads to a poor prognosis. Metastasis occurs slowly, and over 60% of HCC patients are diagnosed at an advanced stage. The molecular evidence indicates that the sudden activation and inactivation of oncogenes and tumor suppressor genes, respectively, lead to hepatocarcinogenesis. The identification of other molecular mechanisms and reliable markers are still needed to detect HCC at the early stages of development [19].

The liver is made up of macrophages, T-cells, cholangiocytes, hematopoietic stem cells (HSC), liver sinusoidal endothelial cells (LSECs), hepatocytes, and neutrophils that are highly organized on the acinus proto-central axis. The axis is mainly lined by hepatocytes that constitute a functional unit of study under disease conditions. Liver zonation is observed as a function of the hepatocyte variation along this axis. scRNA-seq’s first application was a liver zonation study in both mice and humans. Over the past three years, a surplus of liver scRNA-seq studies has been published [20,21,22]. A combination strategy of scRNA-seq and smRNA-FISH (Fluorescence in situ hybridization) was applied to obtain spatial information on liver zonation [23]. The major challenge of integrating each cell’s RNA data with spatial information was addressed by using bioinformatics protocol and following specific sequencing strategies. The scRNA-seq method disclosed unimaginable gene expression gradients in different cell lineages along the sinusoid, demarking hepatocytes of the portal (E-cadherin markers; ECAD) and central (cytochrome P450 2E1; CYP2E1) zones, LSECs of the central zone (mast/stem cell growth factor receptor) [24] and HSCs of the portal zone expressing the tumor necrosis factor receptor (TNFR) superfamily member [20]. These data have been used to study mouse liver zonation using immunohistochemistry [23]. The gene expression patterns of the normal homeostatic environment can be compared with HCC disease development conditions.

Li and his coworkers’ experimental data with cluster analysis, singleR, cell marker, pseudotime analysis, and finally Gene ontology and Kyoto encyclopedia of genes and genomes enrichment analysis provided the key references for the HCC clinical diagnosis and prognosis. SCT of 21 HCC patients and 256 normal persons liver samples were collected from the database Gene expression omnibus (GEO). The cells were grouped, marker genes such as the Hub genes prospective regulatory mechanism were identified, explored, and its evolution process was defined. Lastly, the cancer genome atlas database was used to investigate the differential expression pattern of the 10 survival-related hub genes, and it was correlated with HCC patients’ survival and diagnosis [25]. In HCC patients’ ALDOB (glycolytic metabolizing enzyme), APOC3 (Apolipoprotein C gene), APOH (Apolipoprotein H), CYP2E1 and CYP3A4 (members of cytochrome P450 enzyme system), Gc globulin, HRG (Histidine rich glycoprotein), Linc01554 (long intergenic non-protein coding RNA 1554), and pyruvate dehydrogenase kinase 4 (PDK4) being expressed significantly lower compared to normal tissues indicates poor prognosis while high expression of thioredoxin (TXN) was correlated with carcinogenic effect. Thus, based on single-cell data, the hub genes served as a correlation factor to the survival of HCC patients and further would help in studying the molecular mechanism involved in the evolution of liver cancer [25].

## 4. Tumor Microenvironment Analysis

The most efficient treatment for HCC has been reported to be surgical resection [26]. However, in 2018, the European association of study of the liver has reported 50–70% of cases with a high incidence of relapse, which is a highly painful facet. The HCC tumor ecosystem is considered one of the complex systems with heterogeneous cell types. Understanding the spatiotemporal interactions among the cells in the tumor microenvironment is highly essential for studying the development and prognosis of HCC [27]. Immune checkpoint blockades proved to be effective in many cancer types, but for HCC, the results were unsatisfactory. Recently, sc-RNA seq has been used to profile thousands of cells in early relapse HCC patients. This was compared to the profile of primary tumors. It was observed that regulatory T cells levels were decreased with increased dendritic and infiltrated CD8+T cells in early relapse tumors compared to that of classically exhausted primary HCC. CD8+ T cells exhibited an innate-like low cytotoxic state with overexpressed KLRB1 (CD161) and low clonal expansion, while potent immune response which was revealed by differential gene expression and interaction analyses in relapsed state HCC inhibited dendritic cell antigen presentation. Thus, a comprehensive picture of the TME (tumor microenvironment) divulged by sc-RNA seq was highly beneficial to discover more potential therapeutic strategies for HCC [28].

Over the past few years, SCT methodologies have shown substantial progress in the development of the ideal method. Nearly hundreds of methods have been designed, and a specific method is applied depending upon the research objective of the sequence [13]. Few of the different types of single-cell sequencing methodologies applied in Hepatocellular carcinoma have been tabulated (Table 1).

MacParland et al. revealed a human liver single-cell transcriptomics map with 20 distinct cell populations highlighting the presence of intrahepatic discrete macrophage populations using 10× chromium technology [34]. The liver cellular composition has been poorly defined until now, but scRNA-seq studies have aided the construction of the Human liver atlas (HLA) from 10,000 cells of nine human normal liver donors. The study has revealed heterogenous EPCAM+ (Epithelial cell adhesion molecule) populations, cholangiocytes, and TROP2int populations with high potential to form bipotent organoids. The HLA has also revealed phenotypic changes and allowed for the discovery of distinct cell types in normal and HCC livers using CEL-Seq2 technology [35].

One of the costliest protocols, which limits scRNA-seq wide application, is Smart-seq-2. Smart-seq-2 applies template-switching technologies for RT and amplification PCR technologies. This enables full-length transcript sequencing, splicing events study, and also allele-specific expression. An alternative to its cost-effective protocol was developed. CEL-Seq2, in which RNA poly(A) tail is captured by UMI and cellular pre-specified barcodes inserted at cDNA synthesis step. This enables cDNA pooling for amplification and, followed by sequencing from different cells, significantly reduces the cost per run. The cellular barcodes help to identify the cell of origin and UMI count and normalization helps to quantify gene expression in every single cell—thus proving the protocol Smart-seq-2 and CEL-Seq2 to be highly sensitive. Compared to all other methods, 10× chromium generated the strongest consistent data by scaling the largest number of single cells in a short period of time. It showed higher sensitivity, and mitochondrial genes were read in higher fractions [36]. Zhang et al. reported that a combination of two scRNA-seq technologies (SMART-seq2 and droplet-based methods) helped to reveal the liver TME, which had hitherto not been clearly defined. SMART-seq2 captures only a small number of cells with full gene coverage while droplet-based methods analyze a large number of cells with limited gene coverage. Thus, combining these two technologies gives an in-depth understanding of the TME immune landscape in HCC. The potential properties of diverse cell types of CD45+, mature forms of LAMP3+ dendritic cell (DC) clusters exhibiting diverse ligands to regulate many lymphocytes with migration potential (tumor to lymph nodes), were observed as quite distinct from normal DCs. Even the SLC40A1 and GPNMB inflammatory roles of tumor-associated macrophages have been established [37]. The above data provide insight into the nature of immune cells in TME, constituting a valuable resource for identifying biomarkers and novel immunotherapy targets for HCC.

The biomarkers associated with hepatocellular carcinoma identified through single-cell sequencing have been tabulated (Table 2). In another interesting study, the Smart-seq2 sequencing method was used to profile the gene expression of isolated single cells from HCC tumor cells and para-tumor tissue. Firstly, heterogeneous subclones were identified in the above samples whose hub-gene-co-network and functional annotations analysis were carried out followed by pseudo-time analysis with regulated transcriptional factor co-networks to determine HCC cellular trajectory. The experiment found upregulated expression of carbohydrate responsive element binding protein also called MLX interacting protein-like (MLXIPL) in cells of HCC which are reported to be associated with the overall poor survival rate of HCC patients. Notably, MLXIPL upregulation promoted HCC tumor proliferation by increasing the rate of glycolysis and inhibiting the expression of proteins associated with apoptosis. These results identified MLXIPL to be a significant biomarker and potential therapeutic target for HCC [38].

**Figure 2:**
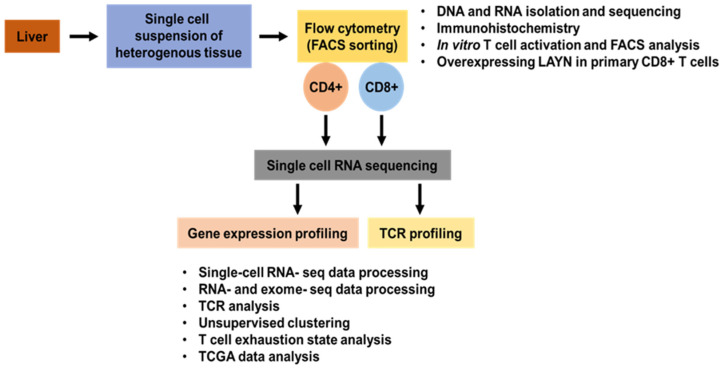
Single-cell technology to study T cells in hepatocellular carcinoma.

Another dynamic scRNA-seq study on the T cell subset population in HCC patients revealed discrete subtypes and characteristics of the tumor-infiltrating T cell landscape. The transcriptional profile of 5063 single T cells isolated from six HCC patients’ peripheral blood, normal and tumorous liver tissues revealed 11 distinct T cell subsets (signature genes of each subset were identified). The subsets were identified based on their similarity to T-cell receptor sequences and molecular and functional properties. Finally, an expression profile study revealed a developmental pathway with connectivity among T cell subsets. Enriched expression and clonal expansion of the T cell subpopulation, such as exhausted CD8+ T cells and infiltrating Tregs, have been observed in HCC. LAYN (Layilin) is one of the genes upregulated in exhausted CD8+ T cells and infiltrating Tregs, but in vitro studies have reported that LAYN’s repress the function of CD8+ T cells. The researchers have compiled a single T cell transcriptome database compendium for the research community to study the characteristics of infiltrating T cells in HCC. They have also developed (http://hcc.cancer-pku.cn, accessed on 25 November 2021) a highly interactive web-based tool to help analyze, visualize, and download the transcriptome data of T cell genes (Figure 2). These comprehensive data provided insights to understand immune TME in HCC [43].

Similarly, Xing and his coworker applied scRNA-seq in an unbiased manner to analyze the microenvironment of the liver. They compared the gene expression pattern in cell populations and subpopulations in HCC and liver fibrosis. Single-cell trajectory analysis results revealed that HCC has greater heterogeneity and the differential gene expression pattern of the epithelial cell subgroups showed unique transcriptional configuration in both HCC and fibrosis. Eight genes were expressed specifically in endothelial and stellate cells of HCC and were correlated with the survival rate of the HCC patients. Five genes (CKS2, MIF, RPL12, HSP90AB1, and S100A6) were highly expressed and three genes (CCL14, CD5L, and APOC3) lowly expressed were associated with shorter survival in HCC patients compared to the low and high expression, respectively, of the same in normal tissues. Thus, this study helped in the identification of new biomarkers for HCC diagnosis and potential treatment targets for HCC therapy [39].

## 5. Single-Cell Genomic Sequencing

Single-cell genome sequencing (sc-genomic-seq) is rapidly advancing in analyzing the complexity of the biological system. It helps in the identification of the location of somatic mutational hotspot areas in genes. Mutation accumulation in this region from birth to centenarians is hypothesized to be the main cause of aging and cancer. Recently, it has been discovered that the Ig genes region in human B lymphocytes is associated with hypermutation hotspots [37]. Sc-genomic-seq is considered to be more challenging than transcriptome sequencing because of the presence of only two copies of DNA compared to thousands of RNA copies per cell. Thus, there is a requirement for whole genome amplification (WGA) before sequencing. The application of this technique is limited because of low accuracy in the detection of variation in copy number and low fidelity. There are two types of WGA techniques based on thermal cycling procedure and isothermal reaction. They are PCR and non-PCR-based technologies. The most commonly applied is degenerative-oligonucleotide-PCR (DOP-PCR) and the non-PCR-based technology includes linear amplification via transposon insertion (LIANTI), and multiple displacement amplification (MDA). Hybrid methods include multiple annealing and looping-based amplification cycles (MALBAC) and PicoPLEX [44]. LIANTI outperforms the above methods. It enables the detection of micro-copy number variation with high resolution at the kilobase level. LIANTI also allows the identification of single nucleotide variations and aids the direct observation of cell to cell differences in the origins of DNA replication stochastic firing. A combination of Tn5 transposition and T7 promoters for in vitro transcription (IVT) is typically used to obtain ample linear amplified transcripts followed by library sequencing. Since LIANTI does not involve exponential amplification, the stability of amplification is greatly enhanced, and spatial resolution is improved [45].

Hybrid methods apply isothermal pre-amplification primarily followed by amplification. Initially added common sequences are amplified by PCR. Compared to the other methods, hybrid technology provides uniformity and intermediate coverage of the genome [46]. In a further study, Duan et al. used sc-WGA sequencing to divulge the relationship between histomorphology and tumor heterogeneity. They studied the profile of 96 liver tumor cells of HBV- associated HCC patients and 15 normal cells and showed that the clonal origin of specific HCC could be either monoclonal or polyclonal (confluent multimodule tumor). In monoclonal HCC, it was observed that variation in copy number occurred in early hepatocarcinogenesis and then remained stable throughout the tumor progression, while the spreading of early intrahepatic clones led to multifocal tumor formation. Notably, ZNF717 was identified as a potential driver gene, exhibiting high-frequency mutation, acting as a tumor suppressor, and regulating the IL-6/STAT3 pathway at both the levels- single and population levels. These results highlight specific tumor evolutionary mechanisms in HCC [40].

DOP-PCR is an efficient method for performing WGA of low-copy genomic DNA. DOP-PCR products are used to genotype insertion or deletion polymorphisms, single nucleotide polymorphism (SNP), and single-stranded conformation polymorphism (SSCP). SSCP is widely used to screen a large number of samples to identify diverse genomic variants in a population of liver cancer cells. It can detect even single point mutations and small sequence variations through the difference in electrophoretic mobility. However, due to a few defects, it is not considered to be optimal for sc-seq. The six base pair random primer used specifically anneals at the 3′ end and amplifies the genome randomly. This results in the loss of information due to low coverage and non-homogenous amplification. Thus, there is a need for a method with low amplification deviation and high fidelity [47]. MDA, a patented method uses random hexamers and phi29 DNA polymerase to generate/amplify unbiased/unlimited genomic DNA. Thus, with wide amplification coverage of the genome, it generates high yields of DNA. Proofreading and 3′-5′ exonuclease activity of phi29 DNA polymerase result in high replication fidelity and low amplification bias compared to DOP-PCR. However, random primers provide uneven genome coverage and amplification bias normally results in a lack of detection and differentiation of alleles in a single cell genome, which, in turn, results in incorrect interpretation of the homozygous and heterozygous loci. Recently, MDA has been used to amplify the genomes of thousands of cancer cells [48,49]. The single-cell epigenetic sequencing (SCES) technique is instrumental in uncovering epigenetic signatures of specific cell types in normal and diseased tissues. Epigenetic profiling also helps to investigate how these cell types and origins develop in tissue and are significantly affected in a disease condition. Recently, single-cell assay transposase-accessible chromatin with sequencing (scATAC-seq) has been used to profile multiple molecular modalities (open chromatin analysis). In the chromatin open regions, sequencing adapters are inserted with the help of transposase, which allows amplification and sequencing of these regions. This gives insight into the role of epigenetic regulation in the progression of different types of cancer and also in brain cell differentiation and maturation [49]. This technique has been extensively applied to create atlases of cell types of disease processes, tissues, and various organisms [50]. From the complex tissue samples, cells are sorted out without applying FACS or magnetic mode of sorting in the sc-ATAC-seq technique. This helps to prevent alteration in the biology of the tissue samples during the isolation process. This technique is widely applied to identify different chromatin accessibility profiles of novel cell subpopulations like cancer stem cells, drug-resistant cells, and infiltrating macrophages in advanced tumors. In a single experiment, thousands of cells’ chromatin accessibility enables to provide insights into developmental trajectories of the cell types. An integrated approach, where scRNA-seq and ATAC-seq were performed along with a partially hepatectomized mice model. This challenge not only mapped the transition state of 13,000 hepatocytes during the liver regeneration process but also uncovered the above mechanism simultaneously performing the key tissue-specific activities [51]. scATAC-seq recently has emerged as a powerful approach as it enabled characterization of gene regulatory activities in HCC. The breakthrough came when chromatin accessibility profiling helped in analyzing transcription regulation (the interaction between cis-acting DNA elements with transcription factor) [52]. Cumulatively, the technique provides a significant reference for the HCC analyses and prognosis. This novel approach aids researchers in easily analyzing changing epigenetic profiles in specific tumor clones and designing epigenetic drugs accordingly. In the future, this approach could be applied to analyze HCC heterogeneity and progression.

## 6. Single-Cell Transcriptomics

Single-cell transcriptomics has scaled the entire human body by developing a Human Cell Atlas (HCA) at single-cell resolution (single-cell atlas). Spatial single-cell transcriptomics is the combination of computational and spatial methodologies that yield data with high resolution and scan genes activities at the cellular level. It is also applied in understanding animals like mice and primates. Recently, it has been widely applied to understand COVID-19 infection by identifying potential sites of viral transmission [53]. These methodologies with improved engineering to map cells of fundamental organs like the liver [54], and in vitro cellular models (organoids) have created wide medical importance. It includes cell therapies, target drug discovery, and regenerative medicine. It has become a valuable platform to create healthy tissue reference maps/atlas and compare it with diseased tissue maps. Genetic variation between the above tissues provides insight into disease mechanisms. This high-throughput technology helps to assess aging progression, gradual disease development, and screening therapeutic response to treatments [54].

It was reported that the scRNA-seq lone approach does not completely diagnose HCC outcome as it does not describe the spatial distribution, it just identifies a subpopulation of cells within the tissue. However, the integration of spatial transcriptomics with this methodology resolves intercellular communication in tumor cells acting in situ. Even hi-plex RNA imaging, multiplexed FISH, and spatial barcoding integration can resolve this issue. However, another report demonstrates that scRNA-seq provides a complete transcriptomic picture underscoring the need for an integrated approach [55].

Liquid biopsy revolutionized hepato-oncology as this helps in the access of tissue samples of the advanced stage HCC patients. Circulating tumor cells’, which are released by the HCC patients in their bloodstream, are the main source of biomarker development. Avola et al. used a genome-wide expression profiling (sequential combination of both flow cytometry and high-density sc-RNA seq) approach to analyze CTC transcriptome heterogeneity and detected overexpression of IGF2 as an oncogenic driver in HCC. Supportively, an anti-tumoral effect was reported in HCC experimental models that were selectively blocked by IGF2 monoclonal antibodies. Remarkably, by just probing tumor mutation landscape, we cannot assess IGF2 downregulation, but, at this juncture, sc-RNA seq of CTC proved to be an outstanding tool in detecting non-mutated genomic aberrations like overexpression of IGF2 [41]. CSCs play a critical role at different stages of tumorigenesis, from initiation to organization of fatal malignancies. Moreover, lncRNAs are reported to be regulating CSCs’ biological function by modulating stem cell-related pathways; its expression (lncRNA HOXA-AS2) in normal cells and HCC cell lines were explored in another pioneering study. The HCC model was developed followed by the determination of its transcriptome profile and stem cell-related markers using high throughput RNA-seq technology combined with single-cell mass cytometry and flow cytometry. It was reported that lncRNA HOXA-AS2 was abnormally upregulated, which plays a pivotal role in the progression of HCC and could be designated as a prognostic biomarker and promising therapeutic target for the treatment of HCC [19].

Yan et al. used single-cell transcriptome analysis to identify the central mechanism triggering the HCC development and progression. Comparison of sc-RNA-seq data of one in vivo tumor cell, two in vitro cell lines, and normal peripheral blood mononuclear cells (PBMCs) was done. They identified proto-oncogene JunB (JUNB), a key component that elicits an immune response and serves a role in HCC development and progression. Integrated results of differential expression analysis—Chromatin immunoprecipitation followed by sequencing (ChIP-seq) and the protein–protein interaction (PPI) network, facilitated the detection of gene regulatory networks and interaction between transcription factors and promoter events. Simultaneously, apolipoprotein A2 (APOA2) with a similar expression pattern as JUNB has been identified as a genetically susceptible protein in HCC. Thus, the study contributed to the identification of novel therapeutic targets to control the progression of HCC [56]. A recent study on single-cell transcriptome analysis exhibited a strong link between ITH (feature specific for tumor aggressiveness) and HCC prognosis. Nearly 56,721 single cells of 46 HCC and intrahepatic cholangiocarcinoma biopsies were analyzed of which 17,164 malignant cells were identified using the above method. Identification of tumor cell evolution players is the significant aspect of the core experimental work. SPP1 was identified as a key candidate, which encodes osteopontin. Osteopontin is a phosphorylated glycoprotein linked with many human diseases. Overexpression of osteopontin is well documented with its pivotal role in HCC progression by regulating different immune cell types in TME [42,57]. Losic et al. integrated three techniques: RNA, DNA, and TCR-sequencing with SNP array data to quantify transcriptomic ITH. Two liver cancer patients’ seven specific regions of 38,553 single cells of the specimen revealed the ITH gene signature. The ITH ecosystem consisted of hepatocytes, cancer-associated fibroblast cells, endothelial, myeloid-derived, and sporadic B-cells. This helped to map or study spatial-temporal interactions between immune and cancer cells. Clonal expansion happening in different regions of the same cancer was also detected. The majority of the cells in clusters were observed to belong to hepatocyte lineage in both of the patients. However, another lineage was observed in the second patient. There was overexpression of GNLY (cytolytic protein secreted to kill tumor cells by activated T cells and NK cells), NKG7, and CCL5 and co-expression of CD3 and GNLY (cytotoxic phenotype). These data clearly state that the first patient HCC cells belong to a less aggressive S3 class compared to the second patient and reveals that TME drives ITH in HCC. These data improved the survival predictions of the patient and elaborated on how components of the heterogenic environment interact during cancer evolution [57]. Single-cell chromatin immunocleavage sequencing (scCHIC-seq) and CoBATCH are high throughputs’ single-cell analysis techniques. These techniques are easier to operate and provide high accuracy, and signal-to-noise ratio with the quality compared to previous technologies [44]. Despite all this advantages, the limitation in the techniques restricts the research and treatment analysis of HCC. These limitations have been overcome by improving technology to get multiomic information at a single cell resolution. 

## 7. Single-Cell Omics Sequencing for Biomarker Development

The single-cell triple omics sequencing (scTrio-seq) technique applies all the three omics sequencing methods–single-cell genome sequencing, DNA methylome, and transcriptome sequencing technologies—on the same single cell. Hou et al. applied this sensitive and reliable technique to identify heterogeneity and complexity within the cell population of the liver biopsied sample, which paved the way for understanding cancer development. It has helped to reveal genetic, epigenetic, and transcriptomic heterogeneity in HCC [58]. Another technique of single-cell multi-omics sequencing (sc-COOL-seq) combines two other techniques for the simultaneous analysis of nucleosome positioning, chromatin state, DNA methylation, nuclear variance, and ploidy in a single cell. Such techniques include nucleosome occupancy, methylome sequencing, and post bisulfite adaptor tagging sequencing. scCOOL-seq helps elucidate the different layers of regulation occurring in both the genetic and epigenetic regions of a particular cell [44].

A combination approach, scRNA-seq along with multi-omics provide necessary information to reconstruct tumor populations subclones’ evolutionary trajectories and resolution analysis at a single cell variant level. The genetic as well as phenotypic heterogeneity information helps to identify functional linkage and defines clonal evolution and principles that regulate cancer drug resistance. Based on mutational profiles of the single cell, Su et al. attempted human liver cancer single variant resolution with a clonal evolution reconstruct based on single-cell mutational profiles [59]. The whole-exome sequencing of five HCC samples revealed major inter-tumor heterogeneity with scRNA seq revealing both genetic and phenotypic heterogeneity. It was interesting to note that different patients’ tumor cells showed gene mutations with a similar expression pattern. The genes analyzed were grouped according to the tissue origin, and the expression pattern was arranged in different panels. These data reported that, in liver cancer, tumor-specific mutations caused phenotypic heterogeneity by modifying the expression of the genes rather than their own ones. These gene expression profiles revealed through bioinformatics data analysis demonstrates which gene is switched on and off, thereby giving clarity as to why specific cancer cells are therapy resistant. This supports oncologic precision medicine in designing new essential therapeutic strategies [59]. In the era of precision medicine, muti-omics (single-cell transcriptomes, proteomes, and epigenetic information) was used to provide multifaceted perceptions into HCC development. Five HCC cell lines (with different metastasis capacity) were analyzed. The integrated approach revealed that the HCC proliferation rate had a negligible role in metastasis, while high mesenchymal status played a pivotal role in strong metastasis capacity. In addition, a hypoxic signature (14 genes of gene set) exhibited by subgroups common in numerous cell lines was identified, which can be related to drug-resistant mechanisms in cell lines. Thus, the results comprehensively provide a better understanding of the molecular mechanism behind the metastasis capacity of HCC, which will guide researchers towards the development of an efficient prognosis for HCC [52].

## 8. Identification of Hepatocellular Carcinoma Origination and Evolution

Identification of HCC origination provides vital information for cancer pathogenesis, initial or early diagnosis, and treatment. Cancer cell origination of melanoma, intestinal, and breast cancer have long been identified and was reported to be the role of progenitor cells, while HCC was reported not to have been originated from progenitor cells but mature hepatocytes. Its high plasticity further complicated the identification of liver cancer origin. However, recently, using SCT, it was found that bone marrow-derived cells (BMDCs) played an important role in HCC origination and evolution [60]. Single cells were isolated from the liver tumor in the DEN-induced HCC model (exhibited histological phenotype and genomic profile similar to human HCCs). Every single cell’s whole genome was amplified using MALBAC. The MALBAC method uses quasi-linear amplification. Primers with eight interchangeable nucleotides and for homogenous hybridization twenty-seven common nucleotides sequences are used. False-positive rates are high due to the common DNA polymerase with proofreading property being used. This can be ruled out by using thermostable or high-fidelity DNA polymerase to generate a microgram of DNA, which can be used for the next-generation sequencing process. Quantitative PCR was used to evaluate amplified DNA products’ genome integrity. Sequencing libraries were constructed using these products (Illumina HiSeq X Ten system). Then, single-cell sequencing data analysis was done, and an ACCTRAN criterion was used to infer phylogeny branch lengths and ancestral character probability distributions. This sequence of experiments indicated the role of BMDCs in mouse HCC formation. However, in humans, it is yet to be confirmed [60].

Over the past few years, SCT has provided information regarding the molecular state of all the individual cells of different cell types in normal liver samples and the thousands of individual cells in tumor tissue biopsy samples. SCT has optimized treatment strategies for metastasis by predicting drug sensitivity, analyzing the mechanism by which a tumor becomes drug-resistant, finding alternatives to overcome tumor resistance, and, finally, understanding relapse after cancer treatment. SCT has emerged as a powerful tool for HCC treatment and is being modified to save time, money, and resources to improve the prognosis of HCC and ultimately liver cancer survival [61].

An interesting study by Navin and his coworkers demonstrated that analyzing multiple cells from the same tumor and single-cell sequencing examining high-resolution copy number profiles aid in understanding the evolution and metastasis of cancer [62]. Furthermore, this method helps in the identification of cell types that were previously undetectable by other approaches. To understand tumor heterogeneity and evolution, 100 single cells from primary breast tumors (52 single cells; monogenomic; genetically homogeneous) and its metastatic liver carcinoma (48 cell nuclei) were analyzed applying single nucleus sequencing. The investigation reported that a single clonal expansion gave rise to the primary tumor and seeded its metastasis. The primary tumor harbored a genetically diverse stagnant copious subpopulation of pseudodiploid cells (diploid peak) and a single subpopulation of aneuploid cells (tetraploid peak), both with low leukocyte infiltration. Among the 24 diploid normal cells of the primary tumor, two cells were reported to have T-cell receptor deletion while not a single pseudodiploid cell was observed among 26 diploid cells from the metastasis. Thus, the emergence of metastatic cells does not take place from an earlier intermediate or a subpopulation but mainly from the advanced expansion. These results were consistent with primary-met pairs deep-sequencing studies indicating that metastatic cells will arise late during the development of the tumor. These results were in contrast to gradual models of tumor progression, which stated that tumor growth takes place as punctuated clonal expansions with very few enduring intermediates [62].

## 9. Limitations and Challenges

scRNA seq has become a powerful technique that empowers the study of complex tumor biology, its heterogeneity, TME architecture, clonal dynamics, and de novo cell types mapping. Due to its sensitivity, a small change in gene expression can drastically influence biological data interpretation. scRNA-seq has emerged as a rapidly evolving technique but still is not applicable for pseudotime analysis [36]. The main challenge faced in scRNA-seq is the complex experimental design. The HCC sample specimens collected cannot be used immediately; they have to be preserved. This is one of the main drawbacks, as the tissue should be preserved intact or dissociated as single-cell suspension (single cell or single nucleus), fixed by methanol or formaldehyde, cryopreserved or live cells, tissue dissociated using trypsin, cold-active protease or a traditional method of digestion at 37 °C. The approaches have been shown to cause artifacts and specific biases which alter the transcriptional profiles of the cell types. Denisenko and his coworkers have done a systematic assessment on kidney tissue dissociation and optimal storage conditions necessary for sc-RNA seq [63,64]. Their data confirmed that digestion on ice avoided stress-related artifacts, cryopreservation resulted in greater loss of epithelial cell types, methanol fixation caused ambient RNA leakage and finally adopting single cell or single nucleus RNA-seq workflows, and the cell type compositions in the specific libraries were not similar [63]. The methods and experimental conditions influenced the cell yield and state of the transcriptomes [64]. Thus, the systematic comparison is extremely necessary to get highly sensitive accurate results. However, systematic assessment for liver sample preservation and tissue processing has not been analyzed yet; in addition, the experimental design performed under different conditions has not been compared with the results of the conventional method. Recently, Michal Slyper’s team studied eight tumor types (including metastatic liver biopsies specimens) to address the above challenges. A systematic toolbox was developed which comprised experimental workflow and methods, computational pipelines, and evaluation metrics. This toolbox will be beneficial for researchers to profile tumors systematically, which will improve precision in both cancer diagnosis and treatment [65].

## 10. Conclusions and Future Perspectives

HCC is a liver cancer characterized by cells exhibiting a unique gene expression profile. Early non-invasive detection is of paramount importance to clinically monitor HCC. Even though the SCT has been proved to be an effective method in cancer diagnosis with the single-cell portal being updated frequently for public reference, it has certain limitations which are overcome by the development of novel computation methods. Single-cell analysis, or transcriptomics, has recently emerged as a powerful tool to detect cancer stem cells or circulating tumor cells at the cellular and genomic scale with respect to ITH. ITH is useful for revealing and investigating tumor metastasis mechanisms and epigenetic alterations, respectively. The result of SCT is a method to help physicians design individual treatment strategies. In other words, SCT helps determine the prognosis of a cancer patient more accurately than ever before. Thus, at the single-cell level, the integration of functional and genomic data analysis uncovers cancer evolution in HCC patients, critical pathophysiological changes, and the development of therapeutic resistance. In the near future, SCT will become a promising novel platform for this liver cancer’s (fatal malignancy) diagnosis and treatment at the individual level, advancing the idea of translational medicine.

## Figures and Tables

**Table 1:** Different types of single-cell sequencing methodologies applied in Hepatocellular carcinoma.

Steps/Technique	Smart-Seq2	CEL-Seq2	10×-Chromium	scATAC-Seq	TARGET-Seq
Cell isolation approach	Low throughput	High throughput	High throughput	High throughput	High throughput
Platform	96/384-well plates; Illumina HiSeq 2000	96/384-well plates; Fluidigm C1, illumine TrueSeq	Drop Seq: Cells with barcoded beads with unique molecule identifiers (UMIs) and primers are used	10× Genomics; Illumina NextSeq 500	Plate-based, Illumina NextSeq 500/550
Measurement	Transcriptome	Transcriptome	Transcriptome	Epigenomics	Genomics
Reverse transcription and c-DNA amplification	Polymerase chain reaction (PCR)	in vitro transcription (IVT). UMI and specific bar codes are used for easy pooling	PCR. UMI and specific bar codes are used for easy pooling	PCR; Barcoded primers	PCR; Barcoded RT primers
Library generation	Tagmentation	Fragmentation	Tagmentation and 3′ enrichment	Tagmentation	Tagmentation
Gene coverage	Full length	3′ part of the gene is sequenced	3′ part of the gene is sequenced	Full length	3′-biased and full length
Sensitivity	High (increasing sensitivity for the detection of low-abundance transcripts, reducing bias towards longer genes, and enabling additional analyses such as assessment of splice variants).	High	High (to quantify individual transcripts, reducing technical noise and amplification bias, but introducing 3′ or 5′ bias depending on the transcript end receiving the tag). Drop-seq exhibits lower capture efficiency and resolution.	Fast and sensitive epigenomic profiling; High variability analysis	High sensitivity, detects multiple mutations in a specific single cell, detects biallelic mutations, detect genomic DNA variants, targeted amplification
Cost	High	Slightly low	Low	High	High
Reference	[29]	[30]	[29]	[31]	[32,33]

**Table 2:** Biomarkers associated with hepatocellular carcinoma were identified through single-cell sequencing.

Biomarker	Expression Pattern	Function	Ref.
MLXIPL	Molecular mechanism of glycolysis activated	Marker exhibits malignant biological behavior by activating glycolysis	[38]
LncRNA HOXA-AS2	High expression	Initiation and progression of HCC	[19]
CKS2, MIF, RPL12, HSP90AB1, and S100A6	High expression	Overall survival rate decreased	[39]
CCL14, CD5L, and APOC3	Low expression	Overall survival rate decreased	[39]
ZNF717	High-frequency mutation	Tumor suppressor activity regulating IL-6/STAT3 pathway	[40]
IGF2	Over expression	Growth regulation	[41]
Osteopontin	Over expression	Potentially regulate different immune cell types in TME; invasion and progression of HCC	[42]

## Data Availability

Not applicable.
